# Genetische Diagnostik bei psychischen Erkrankungen im Erwachsenenalter

**DOI:** 10.1007/s00115-024-01737-y

**Published:** 2024-09-24

**Authors:** Laura L. Kilarski, Isabelle Claus, Elisabeth B. Binder, Franziska Degenhardt, Katharina Domschke, Andreas J. Forstner, Hans J. Grabe, Urs Heilbronner, Daniel Müller, Markus M. Nöthen, Franziska Radtke, Marcella Rietschel, Thomas G. Schulze, Fabian Streit, Ludger Tebartz van Elst, Oliver Tüscher, Jürgen Deckert, Eva C. Schulte, das Referat Genetische, Molekulare, Zelluläre Neurowissenschaften der DGPPN

**Affiliations:** 1 Klinik und Poliklinik für Psychiatrie und Psychotherapie, Universitätsklinikum Bonn, Bonn, Deutschland; 2 Institut für Humangenetik, Universitätsklinikum Bonn, Bonn, Deutschland; 3 Max-Planck-Institut für Psychiatrie, Kraepelinstr. 2–10, 80804 München, Deutschland; 4 Department of Psychiatry and Behavioral Sciences, School of Medicine, Emory University, Atlanta, GA USA; 5 Klinik für Psychiatrie, Psychosomatik und Psychotherapie des Kindes- und Jugendalters, LVR-Universitätsklinikum Essen, Kliniken und Institut der Universität Duisburg-Essen, Essen, Deutschland; 6 Klinik für Psychiatrie und Psychotherapie, Universitätsklinikum Freiburg, Medizinische Fakultät, Albert-Ludwigs-Universität Freiburg, Freiburg, Deutschland; 7 Institut für Neurowissenschaften und Medizin (INM-1), Forschungszentrum Jülich, Jülich, Deutschland; 8 Klinik für Psychiatrie und Psychotherapie der Universitätsmedizin Greifswald, Greifswald, Deutschland; 9 Institut für Psychiatrische Phänomik und Genomik (IPPG), LMU Klinikum, LMU München, München, Deutschland; 10 Centre for Addiction and Mental Health, Campbell Family Mental Health Research Institute, Toronto, ON Kanada; 11 Department of Psychiatry, University of Toronto, Toronto, ON Kanada; 12 Klinik und Poliklinik für Kinder- und Jugendpsychiatrie, Psychosomatik und Psychotherapie, Universitätsklinikum Würzburg, Würzburg, Deutschland; 13 Abteilung für Genetische Epidemiologie in der Psychiatrie, Zentralinstitut für Seelische Gesundheit, Medizinische Fakultät Mannheim, Universität Heidelberg, Mannheim, Deutschland; 14 Department of Psychiatry and Behavioral Sciences, Norton College of Medicine, SUNY Upstate Medical University, Syracuse, NY USA; 15 Abteilung für Psychiatrie und Psychotherapie, Zentralinstitut für Seelische Gesundheit, Medizinische Fakultät Mannheim, Universität Heidelberg, Mannheim, Deutschland; 16 Hector Institut für Künstliche Intelligenz in der Psychiatrie, Zentralinstitut für Seelische Gesundheit, Medizinische Fakultät Mannheim, Universität Heidelberg, Mannheim, Deutschland; 17 Zentrum für Seltene Erkrankungen und Klinik für Psychiatrie und Psychotherapie, Universitätsmedizin Mainz, Mainz, Deutschland; 18 Klinik und Poliklinik für Psychiatrie, Psychosomatik und Psychotherapie, Zentrum für Psychische Gesundheit und Zentrum für Seltene Erkrankungen – Referenzzentrum Nordbayern, Universitätsklinikum Würzburg, Würzburg, Deutschland; 19 DZPG (German Center for Mental Health), Partner Site, München/Augsburg, Deutschland; 20 Department of Psychiatry and Behavioral Sciences, Johns Hopkins University School of Medicine, Baltimore, MD USA

**Keywords:** Genetische Beratung, Psychiatrie, Genetische Syndrome, Komplex-genetisch bedingte psychische Erkrankungen, Genetik psychischer Erkrankungen, Genetic counselling, Psychiatry, Genetic syndromes, Complex genetic mental illness, Mental health genetics

## Abstract

Die vorliegende Arbeit bietet Einblicke in die Rolle genetischer Diagnostik bei psychischen Erkrankungen im Erwachsenenalter. Die Bedeutung genetischer Faktoren in der Entstehung psychischer Erkrankungen und seltener genetischer Syndrome bis hin zu häufigen komplex-genetischen Erkrankungen wird beschrieben. Aktuelle wegweisende klinische Merkmale bei der Indikationsstellung stellen u. a. eine Intelligenzminderung sowie Autismusspektrumstörungen und schwere psychische Erkrankungen mit bestimmten Komorbiditäten wie Organfehlbildungen oder Epilepsien dar. Es wird erläutert, wann genetische Diagnostik leitliniengerecht infrage kommt und in welchen Situationen sie auch ohne Empfehlung in den aktuellen Leitlinien in Erwägung gezogen werden sollte. Es folgt ein Überblick über das Prozedere und derzeit gängige diagnostische Methoden. Aktuelle Limitationen und mögliche Entwicklungen im Bereich genetischer Diagnostik in der Psychiatrie werden diskutiert, inklusive der Tatsache, dass für viele psychische Erkrankungen derzeit keine genetische Diagnostik in der Klinik angestrebt werden soll. Zusammenfassend sollten in der Praxis jedoch in spezifischen klinischen Konstellationen genetische Ursachen mehr in Betracht gezogen und genetische Diagnostik und Beratung angeboten werden.

## Abstract (en)

This review article provides insights into the role of genetic diagnostics in adult mental health disorders. The importance of genetic factors in the development of mental illnesses, from rare genetic syndromes to common complex genetic disorders, is described. Current clinical characteristics that may warrant a genetic diagnostic work-up are highlighted, including intellectual disability, autism spectrum disorders and severe psychiatric conditions with specific comorbidities, such as organ malformations or epilepsy. The review discusses when genetic diagnostics are recommended according to current guidelines as well as situations where they might be considered even in the absence of explicit guideline recommendations. This is followed by an overview of the procedures and the currently used diagnostic methods. Current limitations and possible developments in the field of genetic diagnostics in psychiatry are discussed, including the fact that, for many mental health conditions, genetic testing is not yet part of standard clinical practice; however, in summary genetic causes should be considered more frequently in certain clinical constellations, and genetic diagnostics and counselling should be offered where appropriate.

## Die Rolle der Genetik bei psychischen Erkrankungen

Verhaltensgenetische Studien haben gezeigt, dass ein erheblicher Teil der Genese psychischer Erkrankungen genetisch bedingt ist. Je nach Erkrankung und Alter variiert der Grad der Heritabilität: Bei Depressionen beträgt sie z. B. zwischen 29 und 49 % [20] und bei Schizophrenie ca. 60–80 % [31]. Bei den meisten psychischen Erkrankungen des Erwachsenenalters spielt genetische Diagnostik jedoch im klinischen Alltag bislang keine Rolle. Ausnahmen bilden neurodegenerative Erkrankungen [13] und pharmakogenetische Diagnostik [27], zu welchen bereits separate Übersichtsarbeiten vorliegen [13, 27]. Auch die Rolle genetischer Diagnostik in der Kinder- und Jugendpsychiatrie wurde kürzlich bereits ausführlich dargestellt [4]. Diese Aspekte sind daher nicht Thema der vorliegenden Übersichtsarbeit.

Genetische Syndrome, wie das Fragile-X-Syndrom oder Mikrodeletionssyndrom 22q11.2 (DiGeorge-Syndrom) sind oft mit Entwicklungsstörungen oder Intelligenzminderung vergesellschaftet. Aber auch andere psychiatrische Manifestationen wie Schizophrenie oder Autismusspektrumstörungen (ASS) kommen gehäuft vor [9, 19, 31]. Syndromale Erkrankungen werden aufgrund der Schwere der Symptomatik meist bereits im Kindesalter diagnostiziert [4]. Sie können jedoch bei phänotypisch milden Ausprägungen unerkannt bleiben und sollten bei entsprechenden Hinweisen und Symptomkonstellationen bedacht werden (Tab. 1; [19, 35]).

**Tab. 1:** Eine Auswahl genetischer Syndrome mit psychischer Manifestation

Syndrom	Gen/Region	Häufigkeit in der Allgemeinbevölkerung	Typische somatische Befunde	Mögliche psychische Symptomatik
22q11.2-Deletions-Syndrom	Mikrodeletion 22q11.2	1–3:10.000	Herzfehler, Gaumenanomalien, Hypoparathyreoidismus, Immunschwäche, faziale Dysmorphien, Skoliosen	IQ-Minderung, ASS, ADHS, Schizophrenie, Angststörungen, Depressionen
Angelman-Syndrom	Mutation in *UBE3A*, Maternale Monosomie, Imprinting-Defekt oder uniparentale paternale Disomie 15q11-13	1–2:20.000	Muskelhypotonie, epileptische Anfälle, Ataxie, faziale Dysmorphien, Strabismus	Fehlen expressiver Sprache, ASS, IQ-Minderung, Hyperaktivität, Schlafstörungen
Fragiles-X-Syndrom	CGG-Trinukleotid-Repeat-Expansion in *FMR1*	1:5000 bei Männern, 1:10.000 bei Frauen	Faziale Dysmorphien, Hochwuchs, epileptische Anfälle	ADHS, ASS, IQ-Minderung, soziale Phobie
Klinefelter-Syndrom	Karyotyp 47, XXY	1–2:1000 bei Männern	Hypogonadismus, Hochwuchs	ASS, ADHS, Legasthenie
Neurofibromatose Typ 1 (Morbus Recklinghausen)	Autosomal-dominante Mutationen (95 %) oder Deletionen des *NF1 *Gens	1–5:10.000	(Sub)kutane Neurofibrome, Café-au-lait-Flecken, intertriginöses Freckling, Optikusgliome, Irishamartome (Lisch-Knötchen)	Lernbehinderung, ADHS, Angststörungen, Depression
Prader-Willi-Syndrom	Paternale Deletion, uniparentale maternale Disomie oder Imprinting-Defekt in 15q11-13	1–2:30.000	Kleinwuchs, Hypothyreose, Hypogonadismus, Adipositas	ASS, IQ-Minderung, Hyperphagie, beeinträchtigte Emotionskontrolle und soziale Fähigkeiten
Rett-Syndrom	Mutationen in *MECP2*	1:8500 bei Mädchen	Epileptische Anfälle, Gangataxie, Apraxie, Skoliose, stereotype Handbewegungen	ASS, motorische und sprachliche Regression
Smith-Magenis-Syndrom	Mikrodeletion 17p11.2 oder *RAI1 *Mutation	1–2:25.000	Faziale Dysmorphien, Skelettanomalien, diverse Organfehlbildungen	ASS, Aggressionen, Selbstverletzungen, Schlafstörungen, Entwicklungsverzögerung
Triple-X-Syndrom	Karyotyp 47, XXX	1:900	Epileptische Anfälle, Nierenfehlbildungen, vorzeitige Menopause	Depression, ADHS, Angststörungen, Psychosen, Suizidalität
Tuberöse Sklerose	*TSC1/TSC2*	1–2:25.000	Multisystemische Hamarthome, kortikale Dysplasien, subependymale Noduli, renale Zysten, epileptische Anfälle	ASS, IQ-Minderung, ADHS, Angst- und Zwangsstörungen, selbstverletzendes Verhalten
Williams-Beuren-Syndrom	Mikrodeletion 7q11.23	1:7500	Kardiovaskuläre Erkrankungen, Hyperkalzämie, Diabetes, Osteoporose, Ataxie, Tremor	ADHS, emotionale Dysregulation, Angst- und Schlafstörungen, IQ-Minderung

[4, 7, 9, 30]

Die häufigsten bekannten Syndrome mit Veränderungen in etablierten Krankheitsgenen, Kopienzahlvarianten (CNVs) oder Chromosomenaberrationen und psychischer Komponente sind in Tab. 1 zusammengefasst. Einzeln sind die 11 genannten Syndrome selten, ihre kumulative Häufigkeit liegt jedoch in der Allgemeinbevölkerung bei ca. 0,24 % (Spannweite: 0,17–0,30 %), und es gibt eine Vielzahl weiterer Syndrome mit psychischen Manifestationen [30].

Neben den bekannten genetischen Veränderungen, die den o. g. syndromalen Erkrankungen zugrunde liegen, konnten mithilfe von SNP-Arrays und effizienten Hochdurchsatzsequenziertechniken, dem sog. „next generation sequencing“ (NGS), zusehends weitere seltene genetische Varianten und CNVs identifiziert werden, die im Zusammenhang mit der Entstehung psychischer Erkrankungen stehen.

In einer Studie von 151 erwachsenen psychiatrischen PatientInnen mit „Doppeldiagnosen“ im Sinne einer psychischen Erkrankung UND Entwicklungsstörungen, fazialen Dysmorphien oder ähnlichen Auffälligkeiten konnte bei 45,7 % der PatientInnen eine genetische Diagnose gestellt werden [33]. Es wird angenommen, dass auch bei PatientInnen mit Schizophrenie in 2–3 % und bei ASS in ca. 10 % der Fälle mindestens eine pathologische CNV, bei denen es zu Deletionen oder Duplikation von meist einigen 1000 bis 100.000 DNA-Basen kommt, vorliegt [15, 23, 31, 37]. Durch NGS können die Exone aller Gene („whole exome sequencing“, WES) oder das gesamte Genom („whole genome sequencing“, WGS) sequenziert werden. Bislang wurden z. B. 10 Gene durch WES identifiziert, in denen extrem seltene Varianten odds ratios (ORs) von bis zu 40 erreichen und somit das Risiko einer Schizophrenie signifikant erhöhen [29].

Neueste Arbeiten weisen darauf hin, dass solche genetischen Varianten mit hoher Effektstärke transdiagnostisch sowohl zu einem erhöhten Risiko schizophreniformer Syndrome oder Entwicklungsstörungen als auch neurologischer Erkrankungen wie Epilepsie oder Dystonien beitragen [32]. Auch im Fall familiär vererbter genetischer Veränderungen manifestiert sich die psychische Symptomatik der Familienmitglieder häufig unterschiedlich. Letzten Endes sind die meisten psychischen Erkrankungen, die uns in der Klinik begegnen, auch wenn sie eine prominente genetische Komponente haben, zum einen vielgestaltig und zum anderen multifaktorieller Genese.

Neben seltenen genetischen Veränderungen existieren auch Abermillionen häufiger Einzelnukleotidpolymorphismen („single nucleotide polymorphisms“, SNPs), die im Sinne einer polygenen Vererbung das Erkrankungsrisiko erhöhen können. Eine Vielzahl an SNPs, die mit psychischen Erkrankungen assoziiert sind, konnte bereits mithilfe genomweiter Assoziationsstudien (GWAS) identifiziert werden [2]. Bei der Schizophrenie sind z. B. aktuell SNPs in insgesamt 287 genomischen Regionen („Loci“) mit einem erhöhten Erkrankungsrisiko assoziiert [31]. Auch GWAS bei affektiven Erkrankungen [25, 28] oder der Aufmerksamkeitsdefizit-/Hyperaktivitätsstörung (ADHS) [5] waren bei der Identifikation solcher Risikoloci erfolgreich. Zwischen verschiedenen psychischen Erkrankungen konnten dabei zahlreiche genetische Überschneidungen gefunden werden [16, 21]. Durch Zusammenfassung sehr vieler SNPs eines Individuums im Sinne eines polygenen Risikoscores (PRS) ist es möglich, das kumulative, durch SNPs vermittelte Erkrankungsrisiko abzubilden [2]. Die Varianzaufklärung mittels PRS beträgt bei der Schizophrenie aktuell ca. 9,9 % [31], bei der bipolaren Störung 4,6 % [28] und 1,9 % bei der Major-Depression [20].

Somit decken die derzeit bekannten genetischen Risikofaktoren für psychische Erkrankungen das gesamte Spektrum genetischer Variabilität ab [2, 37] und unterscheiden sich hinsichtlich ihrer Vererbungsmodi, ihrer Effektstärke und Frequenz („minor allele frequency“, MAF) in der Allgemeinbevölkerung. Generell besteht eine inverse Korrelation zwischen Effektstärke und Allelfrequenz einer genetischen Variante [24], sodass bei seltenen Varianten oder CNVs mit einer höheren Penetranz zu rechnen ist.

Über die pathophysiologischen Auswirkungen der meisten krankheitsassoziierten Varianten ist aktuell wenig bekannt, u. a. da die tatsächlich kausale genetische Variante und damit verbundene pathophysiologische Veränderungen oft noch nicht identifiziert worden sind. Mithilfe von Big Data und Omics-Ansätzen (z. B. funktioneller Genomik) wird sich in den nächsten Jahren das Verständnis der molekularbiologischen Grundlagen und Mechanismen der Krankheitsentstehung verbessern [2].

## Für wen kommt genetische Diagnostik infrage?

**Abb. 1:**
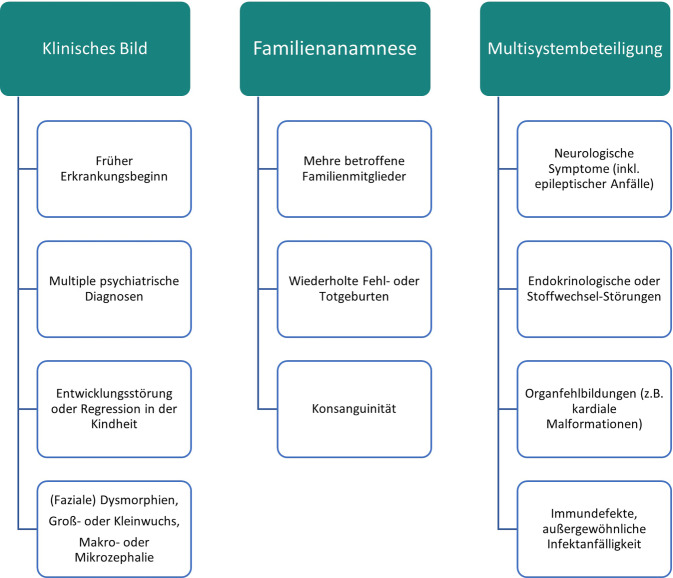
Anamnestische oder diagnostische Hinweise auf eine mögliche genetische Erkrankung

Besondere Merkmale und Befunde, bei denen eine weiterführende psychiatrisch-genetische Vorstellung und Diagnostik sinnvoll sein könnte, sind in Abb. 1 dargestellt. Insbesondere sollte bei atypischem Verlauf einer psychischen Erkrankung (Behandlungsresistenz, früher Beginn, schwerer Verlauf), positiver Familienanamnese, Blutsverwandtschaft der Eltern oder zunächst unzusammenhängenden multisystemischen Symptomen/Erkrankungen an eine mögliche genetische Ätiologie gedacht werden [35]. Eine solche in Betracht ziehen sollte man auch bei Verdacht auf Intelligenzminderung oder anamnestischen Hinweisen auf eine Entwicklungsstörung oder gar Entwicklungsregression im Kindesalter, wie z. B. typisch beim Rett-Syndrom, bei dem es zu einem Verlust bereits erworbener lokomotorischer Fähigkeiten, Verlust der Sprache und der Gebrauchsfähigkeiten der Hände kommt [9, 14].

**Abb. 2:**
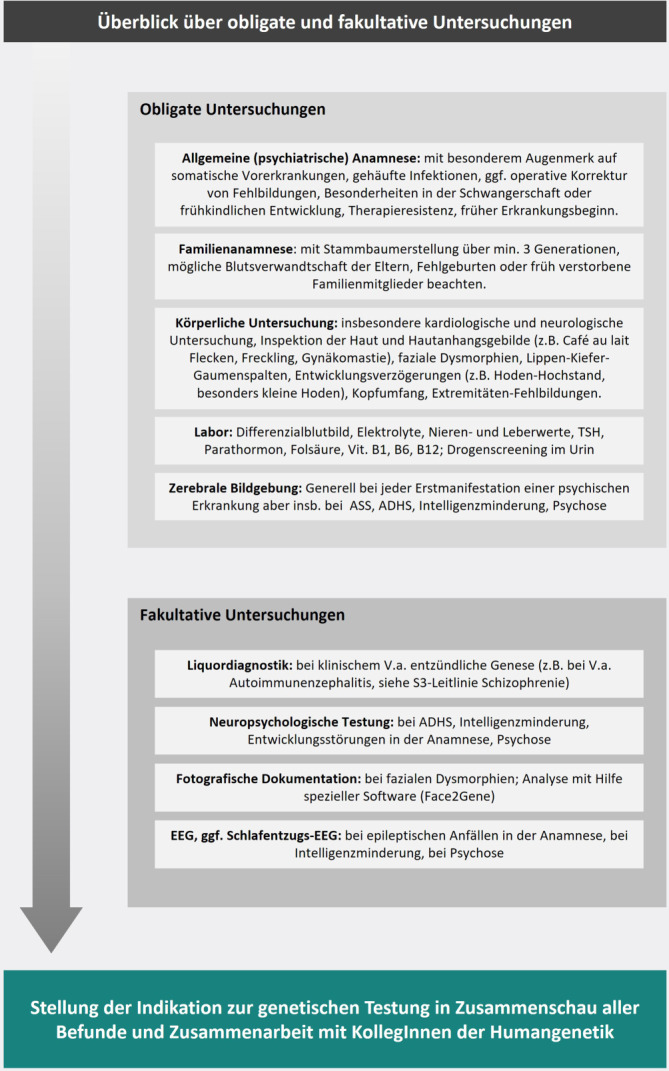
Empfohlene obligate und fakultative Untersuchungen vor der Durchführung einer genetischen Diagnostik

**Abb. 3:**
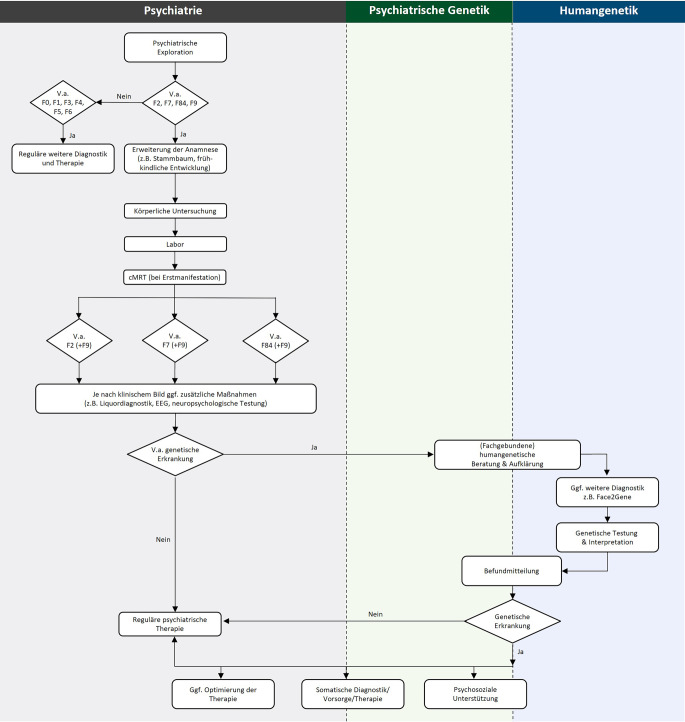
Diagnostischer Algorithmus und Zusammenspiel zwischen Psychiatrie und Humangenetik

Abhängig von der klinischen Präsentation können verschiedene zusätzliche Untersuchungen die Indikation für eine humangenetische Beratung und Diagnostik begründen. Generell sollte eine gründliche Erhebung der Anamnese, psychischer und somatischer Befunde stattfinden, angelehnt an die Empfehlungen der entsprechenden Leitlinien bei Erstmanifestation einer psychischen Erkrankung (Abb. 2). Dies beinhaltet bei klinischem Verdacht auf eine Intelligenzminderung beispielsweise die Verwendung eines standardisierten IQ-Tests [7]. Es sei hier jedoch erwähnt, dass es weder in der Leitlinie noch in der humangenetischen Sprechstunde einen konkreten Cut-off-Wert für den IQ gibt. Entwicklungsstörungen sind von einer Intelligenzminderung abzugrenzen [7] und definiert als eine im Kleinkind- oder Kindesalter deutlich verzögerte oder fehlende Entwicklung bestimmter sprachlicher, kognitiver, emotionaler, sozialer oder motorischer Fähigkeiten [14]. Ein Verdacht auf eine Entwicklungsstörung wird bei Erwachsenen häufig nur noch mittels einer gründlichen (Fremd‑)Anamnese zu erfassen sein, es sei denn es liegen Berichte aus der Kindheit vor. Neben den empfohlenen Untersuchungen in Abb. 2 ist ein möglicher diagnostischer Algorithmus in Abb. 3 skizziert, in dem ebenfalls dargelegt wird, wie der interdisziplinäre Austausch zwischen Psychiatrie und Humangenetik gelingen kann. Hierbei ist selbstverständlich zu beachten, dass bei allen diagnostischen Maßnahmen die Einwilligungsfähigkeit der PatientInnen vorliegen muss oder eine gesetzliche Betreuung mit dem Zuständigkeitsbereich der Gesundheitssorge den Untersuchungen zustimmen muss.

In den nationalen und internationalen Leitlinien und Empfehlungen finden sich aktuell wenig konkrete Angaben zu den Indikationen für eine genetische Diagnostik. Die wichtigsten sollen hier kurz aufgeführt werden.

Die S2k-Leitlinie Intelligenzminderung empfiehlt eine genetische Stufendiagnostik „sofern sich aus Anamnese und körperlicher Untersuchung kein spezifischer V. a. eine ‚nicht-genetische Ätiologie‘ der Intelligenzminderung ergibt“. Hier sei kritisch anzumerken, dass auch bei perinatalen Komplikationen wie z. B. einer Hypoxie unter der Geburt, mögliche genetische Ursachen nicht direkt ausgeschlossen werden sollten. WGS solle bei Personen „durchgeführt werden, bei denen die Ätiologie mit anderen genetischen Verfahren nicht geklärt werden konnte“ [7]. Das britische Royal College of Psychiatrists (RCP) empfiehlt in einem kürzlich publizierten Report eine Chromosomenanalyse, gefolgt von WGS [34].

Laut der deutschen S3-Leitlinie zur ASS soll „eine humangenetische Untersuchung (…) bei bestehender klinischer Indikation (…) empfohlen werden“ [9]. Bei Erwachsenen mit ASS empfiehlt das RCP eine genetische Diagnostik nur, falls Komorbiditäten wie faziale Dysmorphien, kongenitale Fehlbildungen oder weitere komorbide Entwicklungsstörungen bestehen [34].

Die deutschen Leitlinien zu Schizophrenie, ADHS, Depression und bipolar affektiver Störung betonen die komplexe Genese inklusive erblicher Komponente [8, 10, 11, 29]. Empfehlungen zu genetischer Diagnostik finden sich jedoch nicht, wobei sich die Leitlinien für Schizophrenie und ADHS gegenwärtig in Überarbeitung befinden und somit abzuwarten bleibt, ob es in Zukunft konkrete Empfehlungen geben wird. Ein Vorbild kann die S3-Leitinie zu Demenzerkrankungen sein, nach der im Verdachtsfall monogener Ursachen einer Demenzerkrankung genetische Diagnostik (Empfehlungsgrad B) angeboten werden sollte [12]. Empfehlungen zur genetischen Diagnostik werden auch Eingang in die aktuell in Vorbereitung befindliche deutsche S2e-Leitlinie zur apparativen und Labordiagnostik psychischer Erkrankungen finden, die federführend vom Referat für Genetische, Molekulare und Zelluläre Neurowissenschaften der DGPPN erarbeitet werden.

Das RCP empfiehlt bereits bei Psychosen mit komorbiden Entwicklungsstörungen die Testung bekannter CNVs [34]. Eine genetische Diagnostik bei ADHS wird nicht generell empfohlen; wohl aber z. B. bei komorbider IQ-Minderung [34]. Neben den deutschen Leitlinien und dem RCP hat auch die International Society for Psychiatric Genetics (ISPG) ein Statement zur Indikation und Durchführung genetischer Testung bei psychischen Erkrankungen herausgegeben, welches in wesentlichen Punkten kongruent ist [17].

## Weiteres Prozedere und humangenetische Beratung

Während gesetzliche Krankenversicherungen die Kosten für genetische Diagnostik in der Regel übernehmen, sollte bei privat versicherten PatientInnen im Vorfeld eine individuelle Klärung der Kostenübernahme erfolgen. Da genetische Diagnostik im Rahmen ambulanter Behandlungen extrabudgetär erfolgt, sollte sie primär im ambulanten Setting und nicht stationär erfolgen. In der Aufklärung zur genetischen Diagnostik werden mögliche Folgen der Testung aufgezeigt. Zu den Vorteilen zählen eine individuelle Risikoabschätzung insbesondere bei Kinderwunsch und damit mehr Selbstbestimmung in der Familienplanung, mögliche prädiktive Testung weiterer Risikopersonen, mögliche frühere Erkennung und Behandlung von Komorbiditäten durch gezielte Vorsorgeuntersuchungen oder ein besseres Krankheitsverständnis. Sowohl durch Kenntnis des Risikos, aber auch durch Zufallsbefunde oder Varianten unklarer Signifikanz („variants of unknown significance“, VUS) kann jedoch eine psychische Belastung mit vermehrter Sorge um Familienmitglieder oder Angst vor weiteren Erkrankungen entstehen. Betroffene sollen daher dabei unterstützt werden, eigenständig zu entscheiden, ob sie eine genetische Untersuchung wünschen, welche Konsequenzen diese haben könnte oder wie mit Zufallsbefunden zu verfahren ist. Auch auf das Recht auf Nichtwissen muss hingewiesen werden [6, 36].

Approbierte ÄrztInnen (ohne spezifische Qualifikation) dürfen in Deutschland bei symptomatischen PatientInnen, nach entsprechender Aufklärung und schriftlicher Einwilligung, gemäß Gendiagnostikgesetz (GenDG) eine genetische Diagnostik veranlassen [6]. Es empfiehlt sich, vor Einleitung der Diagnostik Kontakt zum humangenetischen Labor, in dem die Testung durchgeführt werden soll, aufzunehmen. Es kann nach dem Gespräch mit dem/der PatientIn/BetreuerIn auch eine Überweisung in eine Spezialambulanz für psychiatrische Genetik oder ein humangenetisches Institut erfolgen, in der alle weiteren Fragen und die Diagnostik besprochen werden. Die Gesprächsführung sollte sich an den Empfehlungen der S2k-Leitlinie Humangenetische Diagnostik und Genetische Beratung orientieren und beispielsweise nondirektiv erfolgen [6] und es gilt, wie in jedem Aufklärungsgespräch, die Einwilligungsfähigkeit des/der PatientIn zu prüfen. Im Rahmen florider Krankheitsschübe ist die Durchführung einer genetischen Diagnostik zumeist nicht erstrebenswert.

Der Befund wird den anfordernden ÄrztInnen persönlich übermittelt und den PatientInnen von diesen mitgeteilt. Den PatientInnen sollte eine genetische Beratung angeboten werden. Diese kann durch FachärztInnen für Humangenetik oder FachärztInnen, welche die Zusatzqualifikation „Fachgebundene genetische Beratung“ erlangt haben, erfolgen. Eventuelle Konsequenzen hinsichtlich eines bestehenden Kinderwunsches (z. B. bei Erkrankungen, die mit einer bekannten Infertilität einhergehen), der therapeutischen Möglichkeiten/Limitationen (z. B. besteht bei PatientInnen mit Mikrodeletionssyndrom 22q11.2 eine erhöhte Anfallsbereitschaft unter Clozapin [19]) und Notwendigkeit primär präventiver Maßnahmen (z. B. kardiologische Mitbetreuung) oder Ausweitung der (psycho-)sozialen Unterstützung (z. B. Selbsthilfegruppen für spezielle genetische Erkrankungen) werden ebenfalls thematisiert.

Für eine prädiktive Testung klinisch gesunder Personen – z. B. Angehörigen von PatientInnen mit einer nachgewiesenen genetischen Erkrankung – ist nach GenDG eine Beratung durch FachärztInnen für Humangenetik oder FachärztInnen mit o. g. Zusatzqualifikation zwingend notwendig. Eine prädiktive Testung im Kontext psychischer Erkrankungen ist derzeit in der Regel auf einzelne seltene neurodegenerative Syndrome beschränkt.

## Diagnostische Methoden

Aktuell erfolgt in der Humangenetik die Diagnostik in der Regel in einem gestuften Verfahren [6], z. B. wie folgt beim Leitsymptom Intelligenzminderung:

1. Chromosomenanalyse (Karyotypisierung),
2. Testung auf *FMR1*-Repeat-Expansionen mittels PCR (Polymerasekettenreaktion, „polymerase chain reaction“),
3. DNA-Microarray (± SNP-Array),
4. NGS-Panel (bei bestimmten klinischen Präsentationen wie z. B. ASS),
5. WES.

Die Chromosomenanalyse dient dem Nachweis numerischer oder struktureller Chromosomenaberrationen, wie beispielsweise beim Down (Trisomie Chr 21)-, Ullrich-Turner (XO)- oder Klinefelter (XXY)-Syndrom. Teilweise findet noch die Fluoreszenz-in-situ-Hybridisierung (FISH) für die Detektion von Mikrodeletionen wie bei der Chromosom-22q11.2-Mikrodeletion Anwendung. Alternativ können Mikrodeletionen und -duplikationen mit einem DNA-Microarray, z. B. der Array-CGH (CGH = komparative genomische Hybridisierung) detektiert werden. Zusehends werden diese durch SNP-Arrays ergänzt, sodass sich auch kopienzahlneutrale Veränderungen wie z. B. uniparentale Disomien finden lassen.

In der molekulargenetischen Diagnostik lassen sich CGG-Repeat-Expansionen in der 5′-untranslatierten Region des Gens *Fragile X Mental Retardation Protein 1 *(*FMR1*) analysieren, die bei Überschreiten einer kritischen Länge von 200 das Fragile-X-Syndrom verursachen. Bei Verdacht auf ein Prader-Willi-Syndrom wird ein Methylierungstest des Chromosoms 15q11-q13 durchgeführt.

In der NGS-Panel-Diagnostik können Sets vordefinierter Gene für ein bestimmtes Krankheitsbild gezielt beurteilt werden. Diese sind z. B. für ASS oder Angelman/Rett-ähnliche Phänotypen gut etabliert.

Wenn sich mit o. g. Methoden keine kausalen Ursachen finden lassen, wird eine WES-Analyse veranlasst. Hierdurch lassen sich insbesondere monogene, dominant oder rezessiv vererbte Erkrankungen identifizieren. Mittels WES können z. B. seltene Punktmutationen und „Indels“ (kleinere Insertionen/Deletionen) erkannt werden.

Prinzipiell kann ein Teil der diagnostischen Schritte vollständig durch NGS übernommen werden, es ist z. B. möglich auch CNVs und Repeat-Expansionen in NGS-Daten zu analysieren [23]. Daher ist zu erwarten, dass die Bedeutung von WES und WGS in der genetischen Diagnostik psychischer Erkrankungen zunehmen wird.

## Aktuelle Limitationen, Risiken und ethische Aspekte

Genetische Untersuchungen sind z. B. in der Pädiatrie, Onkologie und Neurologie bereits gut etabliert. Trotz bedeutender wissenschaftlicher Fortschritte stößt ihre Anwendung in der Psychiatrie jedoch noch auf Grenzen [33].

Psychische Erkrankungen werden zumeist durch eine Kombination genetischer Faktoren, persönlicher Erfahrungen, Umweltfaktoren und Lebensstil beeinflusst. Zusätzlich sind die meisten psychischen Erkrankungen polygen. Viele verschiedene Gene, die miteinander und mit Umweltfaktoren interagieren und an dieser Schnittstelle über epigenetische Prozesse beeinflusst werden, spielen eine Rolle [38]. Die komplexen Interaktionen sind aktuell noch nicht ausreichend verstanden, um aussagekräftige Vorhersagen über klinische Effekte machen zu können. Insbesondere für aus GWAS bekannte Risikoloci sind auch die ursächlichen Gene und damit verbundene Pathomechanismen der genetischen Veränderungen vielfach unzureichend verstanden. Methodisch können durch den Vergleich von Häufigkeiten bestimmter bekannter genetischer Veränderungen in großen Fall- und Kontrollkollektiven lediglich eine statistische Assoziation zwischen einem Phänotyp und einem genomischen Locus hergestellt werden. Sichere Rückschlüsse zur Kausalität lassen sich hieraus nicht ableiten, und die Effektstärken sind z. B. bei der Schizophrenie mit durchschnittlichen ORs von 1,06 (Range: 1,04–1,23) gering [31], sodass einzelne häufige SNPs auf individueller Ebene keinen diagnostischen Nutzen haben. PRS sind aktuell bei psychischen Erkrankungen aufgrund des relativ geringen Anteils der erklärten Varianz, reduzierter transethnischer Aussagekraft aller PRS und unklarer therapeutischer Konsequenz ebenfalls klinisch noch nicht von Nutzen [2], sondern Gegenstand der Forschung. Auch bei seltenen Varianten, die mit psychischen Erkrankungen in Verbindung gebracht wurden, ist die Kausalität nicht immer belegt und die ORs oft geringer als bei seltenen monogenen Erkrankungen.

Der Umgang mit Zusatzbefunden und VUS, wie sie bei WES oder WGS vorkommen können, steht aktuell zur Diskussion. Es handelt sich z. B. um Prädispositionen für onkologische oder kardiologische Erkrankungen, die weitreichende Konsequenzen für PatientInnen und Angehörige haben können. Das American College for Medical Genetics (ACMG) gibt regelmäßig Vorschläge dazu heraus, welche Varianten als Zusatzbefunde an PatientInnen zurückgemeldet werden sollten [26]. Wichtig ist, dass es sich um sog. „actionable secondary findings“ handeln sollte, bei denen präventive Therapieoptionen bestehen. VUS werden bei psychischen Erkrankungen relevant sein, da hier keine großen klinischen Register existieren und insgesamt wenig Daten zur Genotyp-Phänotyp-Korrelation vorhanden sind, um deren Interpretation zu erleichtern. Es bleibt auch zu diskutieren, wie solche Befunde am besten kommuniziert werden können.

Aktuell existieren keine größeren Studien, die sich der Fragestellung widmen, welchen Einfluss genetische Diagnostik auf die psychosoziale Situation psychisch Erkrankter haben könnte. Genetische Diagnostik, insbesondere in der Psychiatrie, wirft ethische und datenschutzrechtliche Diskussionspunkte auf [3, 33].

Diese Punkte sind in besonders hohem Maße auch bei Direct-to-consumer-Gentests (DTC-Gentests) relevant, die sich – trotz ihres Verbots in Deutschland für medizinische Zwecke (GenDG) über das Internet zugänglich – immer größerer Beliebtheit erfreuen. Wenn PatientInnen mit den Ergebnissen solcher Tests Rat suchen, ist es wichtig u. a. zu vermitteln, dass Datenschutzrisiken bestehen können und DTC-Gentests in ihrer Genauigkeit und Aussagekraft nicht einer genetischen Diagnostik nach GenDG entsprechen. Ergebnisse sollten kritisch geprüft werden und ggf. sollten zusätzliche Untersuchungen in Erwägung gezogen werden. Um Fehlinterpretationen der Ergebnisse durch PatientInnen zu vermeiden, ist eine gründliche Erklärung der Ergebnisse, auch in Bezug auf eventuelle Risikoberechnungen oder Wahrscheinlichkeiten, unumgänglich [1, 18]. Die Frage, wie die dafür benötigten Ressourcen bereitgestellt werden können, bleibt offen. Ressourcen hierfür sollten in Zukunft eingeplant und definiert werden.

Gegenwärtig ist der unmittelbare klinische Nutzen einer genetischen Diagnose (im Gegensatz zu pharmakogenetischen Befunden) für pharmakologische Behandlungsentscheidungen begrenzt. Ausnahmen können hier die syndromalen multisystemischen Erkrankungen bilden, bei denen es in Einzelfällen pharmakologische Therapieoptionen gibt. Bei einem Patienten mit Klinefelter-Syndrom ist z. B. eine hormonelle Substitutionsbehandlung sinnvoll, um Folgen wie Osteoporose und metabolisches Syndrom, aber auch psychischen Symptomen wie Antriebslosigkeit oder Niedergestimmtheit entgegenzuwirken. Zudem bestehen insbesondere bei seltenen Erkrankungen Perspektiven hinsichtlich der Zweitverwendung („repurposing“) bereits bekannter Medikamente. Auch nichtmedikamentöse Behandlungsoptionen, die sich aus einer genetischen Diagnose ergeben, können im Sinne primär präventiver Maßnahmen wie beim Chromosom-22q11.2-Mikrodeletionssyndrom und einer intensivierten psychosozialen Unterstützung von Bedeutung sein.

## Ausblick in die Zukunft

Die vollständige Exomsequenzierung (WES) ist derzeit nur in seltenen Fällen eine Standardleistung der gesetzlichen Krankenkassen, jedoch könnte sich dies im Zuge des GenomDE-Projekts (www.genom.de) ändern, welches zum Ziel hat, WGS in die Regelversorgung einzuführen. Auch auf europäischer Ebene gewinnen WES und WGS zunehmend an klinischer Bedeutung. Im Rahmen der Initiative „Beyond 1 Mio. Genomes“ finanziert die EU bis 2027 die Sequenzierung und Bereitstellung klinischer Daten mehr als 1 Mio. EU-Bürger für wissenschaftliche Zwecke (www.digital-strategy.ec.europa.eu/en/policies/1-million-genomes). Diese Daten werden auch einer Verbesserung der genetischen Diagnostik im Bereich psychischer Erkrankungen zugutekommen.

Zudem können mithilfe größerer GWAS auch in nichteuropäischen Populationen die daraus berechneten PRS in ihrer Aussagekraft verbessert werden. Es bleibt jedoch unklar, ob sich PRS bei psychischen Erkrankungen jemals zur individuellen Risikoabschätzung eignen werden. Perspektivisch könnte die Möglichkeit bestehen, PRS bei differenzialdiagnostischen Überlegungen zu nutzen, z. B. um eine unipolare Depression früher von einer bipolar affektiven Störung mit initial depressiver Episode abzugrenzen [22]. Personen mit einem deutlich erhöhten Erkrankungsrisiko, die von bestimmten Therapieformen, präventiven Maßnahmen oder Lebensstiländerungen profitieren könnten, ließen sich bei ausreichend präziser Berechnung des individuellen Risikos durch PRS gemeinsam mit anderen klinischen oder molekularen Prädiktorvariablen, im Sinne eines präzisionsmedizinischen Ansatzes, identifizieren [1, 2].

Genetische Diagnostik wird im psychiatrischen klinischen Alltag häufig nicht in allen Fällen, in denen sie indiziert wäre, veranlasst. Es gibt derzeit nur einzelne Spezialambulanzen für bestimmte genetische Syndrome wie das Zentrum Deletionssyndrom 22q11.2 (ZEDE22q11) des Uniklinikums Würzburg oder universitäre Spezialambulanzen für genetische Diagnostik, z. B. in Bonn und Essen. Somit ist besonders wichtig, dass auch niedergelassene PsychiaterInnen die Genetik im Hinterkopf behalten.

Inwieweit genetische Diagnostik über die oben beschriebenen Szenarien hinaus in der Routinebehandlung psychisch erkrankter Menschen Fuß fassen wird, wird sich in den nächsten Jahren zeigen. Insgesamt fehlen momentan epidemiologische Studien zur genauen Schätzung der Prävalenz genetischer Syndrome bei Menschen mit psychischen Erkrankungen sowie zwingend notwendige prospektive, multizentrische, randomisierte Studien, um den Nutzen weitläufiger genetischer Diagnostik in der Psychiatrie zu evaluieren.

## Fazit für die Praxis

- Aktuell bleibt genetische Diagnostik speziellen Fällen vorbehalten; die Prävalenz dieser ist in der Klinik jedoch wahrscheinlich höher als bisher angenommen.
- Genetische Veränderungen (häufige SNPs, seltene Varianten, monogene Syndrome, CNVs, Mikrodeletionen/-duplikationen und Chromosomenaberrationen) können sich psychopathologisch auf verschiedenste Art und Weise manifestieren.
- Genetische Diagnostik ist indiziert bei ASS, Intelligenzminderung und schweren psychischen Erkrankungen mit bestimmten Komorbiditäten.
- Enge Zusammenarbeit zwischen Psychiatrie und Humangenetik ist zielführend.

## Abkürzungen

ACMG: American College for Medical Genetics
ADHS: Aufmerksamkeitsdefizit‑/Hyperaktivitätsstörung
ASS: Autismusspektrumstörung
CNV: „Copy number variant“ – Kopienzahlvarianten
CGH: „Comparative genomic hybridization“ – komparative genomische Hybridisierung
DTC-Gentests: Direct-to-Consumer-Gentests
FISH: Fluoreszenz-in-situ-Hybridisierung
FMR1: Fragile X Mental Retardation Protein 1
GenDG: Gendiagnostikgesetz
GWAS: Genomweite Assoziationsstudie
IQ: Intelligenzquotient
ISPG: International Society for Psychiatric Genetics
MAF: „minor allele frequency“ – Allelfrequenz
NGS: „next generation sequencing“ – Hochdurchsatzsequenzierung
OR: Odds Ratio
PCR: „polymerase chain reaction“ – Polymerase-Kettenreaktion
PRS: „polygenic risk score“ – polygener Risikoscore
RCP: Royal College of Psychiatrists
SNP: „single nucleotide polymorphism“ – Einzelnukleotidpolymorphismen
VUS: „variant of unknown significance“ - Varianten unklarer Signifikanz
WES: „whole exome sequencing“ – vollständige Exomsequenzierung
WGS: „whole genome sequencing“ – vollständige Genomsequenzierung
ZEDE22q11: Zentrum Deletionssyndrom 22q11.2
